# Keratin 17 modulates the immune topography of pancreatic cancer

**DOI:** 10.1186/s12967-024-05252-1

**Published:** 2024-05-10

**Authors:** Lyanne Delgado-Coka, Michael Horowitz, Mariana Torrente-Goncalves, Lucia Roa-Peña, Cindy V. Leiton, Mahmudul Hasan, Sruthi Babu, Danielle Fassler, Jaymie Oentoro, Ji-Dong K Bai, Emanuel F. Petricoin, Lynn M. Matrisian, Edik Matthew Blais, Natalia Marchenko, Felicia D. Allard, Wei Jiang, Brent Larson, Andrew Hendifar, Chao Chen, Shahira Abousamra, Dimitris Samaras, Tahsin Kurc, Joel Saltz, Luisa F. Escobar-Hoyos, Kenneth R. Shroyer

**Affiliations:** 1 Department of Pathology, Renaissance School of Medicine, Stony Brook University, 101 Nicolls Road, Stony Brook, NY 11794 USA; 2 Program of Public Health and Department of Preventative Medicine, Renaissance School of Medicine, Stony Brook University, Stony Brook, NY USA; 3 Department of Pathology, School of Medicine, Universidad Nacional de Colombia, Bogotá, Colombia; 4 Department of Computer Science, Stony Brook University, Stony Brook, NY USA; 5 Center for Applied Proteomics and Molecular Medicine, George Mason University, Fairfax, VA USA; 6 Perthera, McLean, VA USA; 7 Scientific and Medical Affairs, Pancreatic Cancer Action Network, Manhattan Beach, CA USA; 8 Department of Pathology, University of Arkansas for Medical Sciences, Little Rock, AR USA; 9 Department of Pathology and Genomic Medicine, Sidney Kimmel Cancer Center, Thomas Jefferson University Hospital, Philadelphia, PA USA; 10 Departments of Pathology and Medicine, Cedars-Sinai Medical Center, Los Angeles, CA USA; 11 Department of Biomedical Informatics, Renaissance School of Medicine, Stony Brook University, Stony Brook, NY USA; 12 Department of Therapeutic Radiology, Yale University, New Haven, CT USA; 13 Department of Molecular Biophysics and Biochemistry, Yale University, New Haven, CT USA; 14 Division of Oncology, Department of Medicine, Yale University, New Haven, CT USA

**Keywords:** Keratin 17, Pancreatic ductal adenocarcinoma, Cancer immunology, Cancer biomarker, Immune microenvironment, Multiplexed immunohistochemistry, Digital pathology

## Abstract

**Background:**

The immune microenvironment impacts tumor growth, invasion, metastasis, and patient survival and may provide opportunities for therapeutic intervention in pancreatic ductal adenocarcinoma (PDAC). Although never studied as a potential modulator of the immune response in most cancers, Keratin 17 (K17), a biomarker of the most aggressive (basal) molecular subtype of PDAC, is intimately involved in the histogenesis of the immune response in psoriasis, basal cell carcinoma, and cervical squamous cell carcinoma. Thus, we hypothesized that K17 expression could also impact the immune cell response in PDAC, and that uncovering this relationship could provide insight to guide the development of immunotherapeutic opportunities to extend patient survival.

**Methods:**

Multiplex immunohistochemistry (mIHC) and automated image analysis based on novel computational imaging technology were used to decipher the abundance and spatial distribution of T cells, macrophages, and tumor cells, relative to K17 expression in 235 PDACs.

**Results:**

K17 expression had profound effects on the exclusion of intratumoral CD8+ T cells and was also associated with decreased numbers of peritumoral CD8+ T cells, CD16+ macrophages, and CD163+ macrophages (p < 0.0001). The differences in the intratumor and peritumoral CD8+ T cell abundance were not impacted by neoadjuvant therapy, tumor stage, grade, lymph node status, histologic subtype, nor KRAS, p53, SMAD4, or CDKN2A mutations.

**Conclusions:**

Thus, K17 expression correlates with major differences in the immune microenvironment that are independent of any tested clinicopathologic or tumor intrinsic variables, suggesting that targeting K17-mediated immune effects on the immune system could restore the innate immunologic response to PDAC and might provide novel opportunities to restore immunotherapeutic approaches for this most deadly form of cancer.

**Supplementary Information:**

The online version contains supplementary material available at 10.1186/s12967-024-05252-1.

## Introduction

Pancreatic ductal adenocarcinoma (PDAC) is one of the most lethal forms of cancer, not only because it is often not diagnosed until after it has reached advanced stage and is intrinsically resistant to Gemcitabine and 5-fluorouracil based chemotherapy, but because it generally does not respond to immune checkpoint inhibitors and is minimally impacted by intrinsic anti-tumor immune mechanisms [31]. Although immune evasion is a key hallmark of malignancy, impacting cancer initiation and progression, knowledge of the mechanisms that shield PDAC from immune surveillance have not been fully explored. Therefore, elucidation of the interactions between PDAC and the immune response is critically needed to guide the development of more effective immunotherapeutic strategies.

Several studies have stratified PDAC patients into separate categories through transcriptomics, proteomic analysis, gene signatures or immunological status using bulk RNA-Seq, immunohistochemical, and single-cell RNA (scRNA) approaches [9, 13, 64]. Although numerous transcriptomic and proteomic reports have shown that PDAC can be subdivided into major molecular subtypes that differ in response to chemotherapeutic agents and patient survival, little is known about how biologically distinct PDACs can differ in their immunogenic phenotypes, or the impact of the immune response on disease progression and survival. To the best of our knowledge, this is the first study that aims to consolidate the histological subtype stratification with the tumoral microenvironment status to better understand tumor aggression and rationalize more personalized therapeutic strategies. We and others have shown that keratin 17 (K17) drives chemoresistance and is a prognostic and predictive biomarker of the most aggressive (basal) molecular subtype of PDAC [35, 37, 38, 46, 47]. K17 expression also impacts the immune response in several cancer types, including basal cell carcinoma, head and neck cancer [14, 57, 58], and cervical squamous cell carcinoma [5]. At a mechanistic level, K17 has also been reported to impact the pathogenesis of cervical squamous cell carcinoma, at least in part via immunomodulatory mechanisms [59] and others have explored mechanisms through which K17 might regulate resistance to immunotherapy, through the regulation of Yap1 activation, mediating downstream immunosuppressive effects in head and neck cancer. The potential impact of K17 on the immune response to PDAC, however, has not been previously explored.

Thus, it is important to consolidate different stratification schemes into a novel classification of pancreatic cancer, based on robust and clinically deployable biomarkers to predict survival and to rationalize therapeutic strategies. Several studies have emphasized the importance of cancer cell clearing by intratumoral and peritumoral immune cells, with favorable prognosis related to the extent of intratumoral immune infiltration [43]. Since successful immunotherapy is dependent on the infiltration into the tumor of sufficient effector cells, including CD8+ T cells and tumor-associated macrophages, we aimed to characterize the PDAC immune microenvironment relative to K17 expression by focusing on peritumoral and intratumoral immune cells via a comprehensive, distance-based spatial analysis using brightfield multiplex immunohistochemistry (mIHC) of PDAC tissue sections. Overall, these lines of exploration may uncover how tumor cell-intrinsic immunomodulatory proteins, including K17, may shield PDAC from the development of effective immune responses and may highlight opportunities for further exploration to develop novel and more effective immunotherapeutic approaches for PDAC.

## Methods

### Patient demographics

Primary PDAC surgical resection specimens (n = 235) were provided as formalin-fixed paraffin-embedded (FFPE) surgical tissue blocks from the archival collections of the Department of Pathology at Stony Brook University Hospital (n = 54, 23%), Thomas Jefferson University (n = 67, 29%), Cedars Sinai Medical Center (n = 7, 3%) and a national biorepository, the Know Your Tumor program of the Pancreatic Cancer Action Network (PanCAN/Perthera) (n = 107, 45%).

Hematoxylin and eosin-stained sections from each specimen were reviewed to identify the single tissue block that contained the greatest total surface area of viable carcinoma. Exclusion criteria included cases where the total surface area of viable tumor was < 1 cm^2^. Additionally, tumors metastatic to the pancreas from other anatomic sites were also excluded. Survival and adjuvant therapy data was obtained from each respective institution’s registry. Case stratification was based on tumor stage, histologic subtype, and histologic grade. Tumor stage was assigned based on 8th edition American Joint Committee on Cancer (AJCC) criteria [3, 11] and histopathologic grade was based on World Health Organization (WHO) criteria [33]. Table 1 summarizes the demographic and clinicopathologic features of all cases. Missense mutations, copy number alterations (CNA), truncations, splicing events, and fusions of KRAS, p53, SMAD4, and CDKN2A were tested for potential correlation to CD4+, CD8+, CD16+, and CD163+ immune cell infiltrates. Missense mutations were classified as tolerant versus deleterious using the Sorting Intolerant From Tolerant (SIFT) tool, which predicts the impact of single amino acid substitutions [34].

**Table 1:** Patient cohort demographics

	Cohort
Total cases included	n = 235
Overall survival, mean ± SD	20.7 ± 17.1
Age at diagnosis, mean ± SD	62.9 ± 14.3
Gender, number (%)	
Female	109 (46%)
Male	123 (52%)
Unknown	3 (2%)
Histologic grade (G), number (%)	
G1 + G2, well and moderately differentiated	172 (73%)
G3, poorly differentiated	63 (27%)
AJCC 8th edition pathological stage, number (%)	
I-IIB (early)	65 (27%)
III-IV (advanced)	165 (70%)
Unknown	5 (3%)
Chemotherapy	
Neoadjuvant	41 (17%)
No neoadjuvant	194 (83%)
Histologic subtypes	
Conventional	180 (77%)
Foamy gland [2]	20 (9%)
Large duct [50]	18 (8%)
Other	17 (6%)
Genetic mutation status	
KRAS, p53, SMAD4, CDKN2A	90 (38%)
Availability of survival information, number (%)	
Number of cases	219 (93%)
Unavailable	16 (7%)

### mIHC

Multiplexed immunohistochemistry (mIHC) was performed on 5 µm formalin-fixed paraffin-embedded tissues using a Discovery Ultra Automated Slide Staining System (Roche, Oro Valley, AZ). Briefly, slides were baked at 60° for 32 min and deparaffinized at 70 C for 8 min for 3 cycles. Antigen retrieval was performed for 64 min at 100 C using TRIS–EDTA buffer (Ventana Medical Systems, Catalog #: 950-500). Slides were then treated with Discovery inhibitor (Roche, Catalog #: 760-4840) for 8 min to block endogenous peroxidase activity and blocking performed with S Block (Roche, Catalog #: 760-4212) for 8 min. Slides were stained sequentially with primary antibody, linking antibody, enzyme-conjugated antibody, and chromogen. After each round of staining, antibody complexes were removed using CC2 (Roche), a pH 6.0 citrate/acetate-based buffer containing 0.3% SDS, and heating the slide to 93 degrees for 8 min. Antibodies for CD4 (helper T cells), CD8 (effector T cells), CD16 (pan-macrophage), CD163 (M2 macrophage), pancytokeratin (panCK), and K17 were provided by Roche Diagnostics Corporation through a sponsored research agreement (RD005216). Multiple chromogens (Red: CD4+, Purple: CD8+, Yellow: CD16+, CD163−, Green: CD16+, CD163+, Teal: panCK+, and Brown: K17+) were deployed to enable multispectral imaging of diverse immune cell populations within the cancer microenvironment. Details of the mIHC protocol are outlined in Supplementary Table 1.

### Optimization of mIHC protocol

mIHC conditions were optimized and validated using tissue controls for each individual antibodies. Serial sections of PDAC and tonsil cases were stained with individual antibodies. Upon slide review by a board-certified clinical pathologist, quality of staining, color intensity, and patterns of IHC staining was assessed for each antibody before inclusion on the mIHC sequence protocol. In addition, we ran negative controls that substituted diluent for each of the primary antibodies and secondary antibodies. Additionally, sensitivity of the antigens to repeated denaturation steps was evaluated by running several staining protocols and localizing the primary antibody in different locations within the sequence and adding multiple denaturation steps before or after as needed to account for denaturations in the full mIHC protocol. Antigens that were sensitive to repeated denaturation were placed later in the sequence.

### Whole slide image acquisition

All slides were reviewed by a board-certified clinical pathologist and regions of interest (ROIs) drawn around tumor areas and to exclude areas of pancreatitis, large areas of necrosis, and normal pancreas. An Olympus VS120 microscope (Olympus, Tokyo, Japan) was used to scan glass slides and generate digital whole slide images (WSIs) at 20× magnification with a resolution of 0.346 μm per pixel. Scanned bright field images were annotated using Quantitative Imaging in Pathology (QuIP) Software [49]. Pathologist-defined ROIs were transposed onto scanned mIHC images and supervised image classification, and output applications were subsequently performed.

### Cell detection and classification

The ensemble of ColorAE and U-Net were previously developed for the detection and classification of cells in mIHC images based on the decomposition of mIHC images into their constituent stain maps, where the dominant stain at each location indicates the corresponding biomarker and hence the corresponding cell type [17, 20]. ColorAE is a deep autoencoder which segments stained objects based on color, U-Net is a convolutional neural network (CNN) trained to segment cells based on color, texture and shape. The two methods provide complementary information and are used together to predict K17 positive and negative cells as well as four immune cell types [20]. Each model is trained separately, and predictions from each model are combined in the inference phase to create multi-class masks. The multiplex segmentation ensemble is applied on patches of size 580 × 580 pixels extracted from whole slide images (WSIs) at 0.346 μm/pixel resolution. The spatial analysis is applied on patches extracted from tumor bed regions that were manually annotated by two pathologists as depicted in Supplementary Fig. 1. Multi-class masks are generated on patches using ColorAE and U-Net as described [20].

### Dataset description

The Training and Validation datasets were generated from 23 randomly chosen whole slide images (WSIs). From these 23 WSIs, 57 1000 × 1000 pixels ROIs were selected for training and four 1000 × 1000 pixels ROIs were selected for validation. Training and validation datasets were derived from different sets of WSIs. The Testing dataset was generated from 8 randomly chosen WSIs (disjoint from the training and validation WSIs), and 32 400 × 400 ROIs were selected from these 8 WSIs. We augmented the training data and validation data by extracting 16 overlapping 400 × 400 patches from each ROI. In summary, we used 912 400 × 400 overlapping patches for training, 64 400 × 400 overlapping patches for validation and 32 400 × 400 non-overlapping patches for testing. In addition, two pathologists independently carried out an extensive qualitative evaluation of 60 ROIs drawn from more than 20 slides. For the training and evaluation, we use weak labels in the form of dot annotations performed by independent pathologists (Supplementary Fig. 1). The dot annotations are transformed into multi-class superpixel masks using simple linear iterative clustering (SLIC) algorithm [1]. SLIC groups pixels are then transformed into super pixels based on their color and spatial proximity, using a k-means clustering approach. Supplementary Fig. 2 shows an example from a training ROI with its corresponding ground truth annotation (combination superpixel label and dot annotation) overlaid on top of the original image.

### Model validation and experimental setup

We carried out a quantitative evaluation of our detection and classification model as previously described [17, 20]. Briefly, we assessed the performance of our methods using: F1 score, Recall, and Positive Predictive Value (PPV) to evaluate the predictions from ColorAE: U-Net ensemble methods against pathologist-generated ground truth. For our ColorAE: U-Net ensemble model evaluation, we tabulated PPV, Recall, and F1 scores using different dilations of seed in Supplementary Table 2. In summary, we employed positive predictive value, recall, and F1 scores to assess stain detection by comparing predictions against dilated seed labels. The seed labels were dilated into 10 μm diameter disks and 5 μm diameter disks to evaluate the sensitivity of the evaluation scores. Each colored stain mask underwent a separate evaluation. Evaluating true positives (TP), false positives (FP), and false negatives (FN) involved assessing the overlap between the predictions and dilated seed labels. Specifically, TP denotes the number of connected components in the mask overlapping with the dilated disks; FP signifies the number of connected components that lack overlap with any disks; and FN indicates the number of disks without overlap with the mask. TP, FP, and FN values were aggregated across all 6 testing patches to address potential sparse cell types in certain patches. These aggregated values were then utilized to compute the F1 score, precision, and recall. Comparing the F1 scores, precision, and recall for both 10 μm diameter disks and 5 μm diameter disks, we did not detect a high deviation between the scores for any of the immune cell types except for CD4+ T cells. Hyperparameters including color concentration thresholds were selected based on the quantitative and qualitative evaluation of the validation set. Based on our evaluation on validation set, we selected a dropout rate of 0.3 in the U-Net and the following color concentration thresholds in the colorAE model: 0.7 for K17-positive, 0.1 for K17-negative, 0.1 for CD4, 0.1 for CD8, 0.1 for CD16, and 0.1 for CD163. We carried out computation using resources provided through the National Science Foundation digital cyberinfraestructure eXtreme Science and Engineering Discovery Environment (XSEDE) [54].

### Quantification of tumor-immune cell spatial relationships

The tumor regions were partitioned into K17-positive and K17-negative zones, leveraging the masks generated with the ensemble model. Our goal was to compare immune cell density in regions close to K17-positive vs K17-negative tumor zones as well as intra-tumoral immune cell densities. First, we assessed the relative density of stromal immune cells in a range from 5 to 200 µm of the closest tumor border (defined as peritumoral immune cells) versus those that are in direct contact with K17-positive vs K17-negative tumor cells (defined as intratumoral immune cells). Distances were chosen based on the potential to define cell neighbors participating in direct cell–cell contact (25 µm) or close-range signaling (200 µm). As the maximal differences in peritumoral immune cell counts relative to K17 status were seen at a stromal depth of 25 μm the analysis of all cases included in the study was done only at 25 μm (Supp. Figure 3). In a conceptual sense, the approach we took was to associate each immune cell with K17-positive tumor cells when the closest tumor boundary to the cell was K17-positive and to associate immune cells with K17-negative tumor when the closest tumor boundary was K17-negative. The analysis described below formalizes this approach.

A distance transform mapped each pixel to the closest boundary of interest. We only considered stromal immune cells that are within 25 μm of the closest tumor boundary; this region was computed using the distance transform [16, 52]. We then partitioned this tumor-associated stromal region into K17-positive and K17-negative zones, leveraging the distance transform field of the stromal area. A stroma pixel was assigned to the K17-positive influence area when the closest tumor boundary was K17-positive, according to distance transform calculation; otherwise, the pixel was assigned to the K17-negative influence area. We devise a metric that we named the “Tumor/Stromal Zone Score”, denoted by ${ZS}_{M}^{ic}$, calculated by the following equation:

$${ZS}_{M}^{ic}=\frac{{Cell \; Count}_{M}^{{i}_{c}}}{{Tumor/Stroma\; Zone}_{M}}.$$

In the equation, ${i}_{c}$ represents immune cells (e.g., CD4, CD8, CD16 and CD163), and M represents the marker of tumor nest boundary (e.g., K17-positive boundary and K17-negative boundary). ${Cell\; Count}_{M}^{ic}$ represents the number of immune cells of type ${i}_{c}$ in either a K17 positive zone or in a K17 negative zone. The equation represents the approximate count of each immune cell (numerator) normalized by the total tumor-associated stromal zones (denominator). The estimation of immune cell count is achieved through a series of steps, commencing with the computation of total pixel numbers specific to distinct cellular subtypes. Following this, the pixel measurements were converted into square micron area units, subsequently undergoing normalization based on the average dimensions of immune cells. Notably, lymphocytes (CD4, CD8) average dimensions were approximated as circles with a diameter of around 8 µm, while macrophages (CD16, CD163) average dimensions are approximated as circles with a larger diameter of 16 µm. This normalization process culminated in the derivation of an estimated count of immune cells, designated as ${Cell\; Count}_{M}^{ic}$ and represented in the Equation. In addition to calculating Tumor/Stroma Zone Score, we normalized Tumor/Stromal Zone Score for K17-negative ($Z{S}_{K17-}^{ic}$) with respect to Tumor/Stromal Zone Score for K17-positive ($Z{S}_{K17+}^{ic}$) for visualization and interpretation purposes, as depicted in multiple figures. Lastly, we performed proof of concept demonstrating that our observed pattern for Tumor/Stroma zone score for all the WSI is not random. We tested a statistical null hypothesis by randomly placing simulated immune cells in the tumor microenvironment and observed a statistically significant difference between the real and simulated scenarios, as previously reported [20].

### Statistical analysis

Paired t tests were performed to define the difference between peritumoral and intratumoral immune cell counts in K17 positive and K17 negative regions of each case. Statistical significance was set at p-value ≤ 0.05, and analysis was done using SAS 9.4 (SAS Institute, Cary, NC, USA) and Graph Pad Prism 7 (Graph Pad Software, La Jolla, CA, USA). All p values were calculated using a two-sided test.

## Results

### Quantification of tumor-immune cell spatial relationships model

As the immune system is known to have a crucial role in cancer and play an essential role in eradicating tumor cells, the characterization of the immune component of the tumor microenvironment (TME) can provide valuable information regarding the ways in which the host immune response interacts with cancer cells [23]. We deployed mIHC and machine-learning tools to quantify T cells and macrophages in the tumor microenvironment relative to K17 expression by tumor cells across a broad range of clinically diverse PDAC cases (Supp. Figure 4).

### Overall immune cell landscape in PDAC

**Fig. 1:**
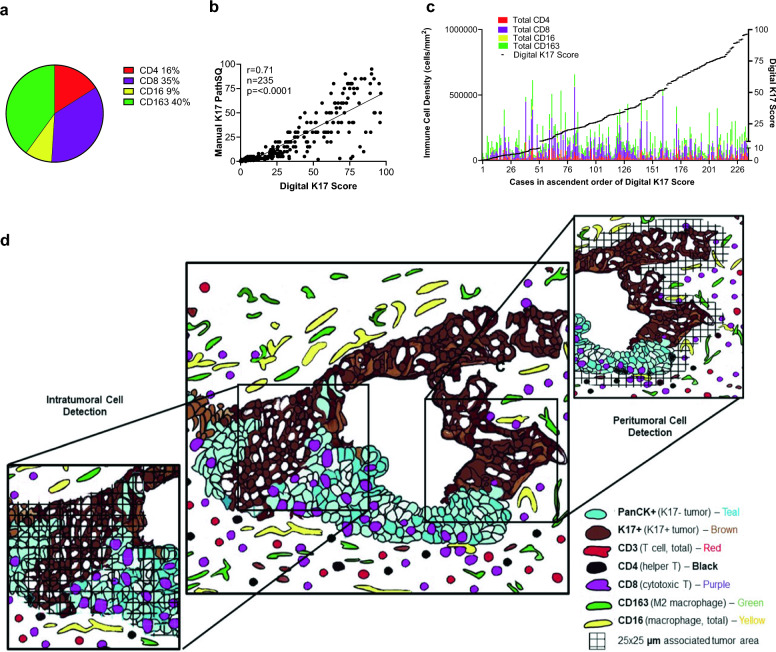
Analysis of Keratin 17 relative to the PDAC immune microenvironment. **a** Overall fraction of immune cell types averaged across all cases (n = 235). **b** Spearman correlation between manual and digital K17 scoring across entire tumor sections. **c.** Overall immune cells stacked bar plot including CD4+ T cells, CD8+ T cells, CD16+ macrophage, and CD163+ macrophage density (cells/mm^2^). The right Y-axis depicts the overall K17 score within each tumor. **d** Development of a digital scoring system focused on spatial relationships between peritumoral and intratumoral immune cells and K17. Intratumoral zones were defined as those that directly contacted a tumor cell while peritumoral zones included only immune cells within 25 μm of the closest tumor cell boundary

The immune populations of 235 PDAC patients were processed by mIHC for a panel of myeloid and lymphoid cell markers encompassing CD8+ T cells, CD4+ T cells, CD16+/CD163− (M1) macrophages and CD16+/CD163+ (M2) tumor-promoting macrophages. Based on overall cell counts across all cases, 16% of immune cells were CD4+ T cells, 35% were CD8+ T cells, 40% were CD163+ macrophages, and 16% were CD16+ macrophages (respective mean counts 1.04 × 10^4^/µm^2^, 3.00 × 10^4^/µm^2^, 3.03 × 10^3^/µm^2^ and 2.44 × 10^4^/µm^2^) (Fig. 1a). We confirmed that K17 is a negative prognostic biomarker by IHC with the available survival information from patients included in this study (n = 219), applied the same threshold (10%) and confirmed that high levels of K17 was a negative prognostic biomarker, with a median survival of 22.1 months for high K17 cases (HR = 1.548, P = 0.0391) compared to those in the low K17 expression group (median = 36.2 months) (Supp. Figure 5). To determine if the immune microenvironment was correlated with K17 status and to verify the accuracy of digital score, we confirmed that the K17 status based on a semi-quantitative manual scoring within a single representative histologic section from each case to K17 scoring based on image analysis of corresponding whole slide digital images (r = 0.71, p < 0.0001) (Fig. 1b). We then tested for correlations between the overall digital K17 score derived each tissue section to the immune cell counts for each case. Sorting patient’s immune densities in ascendant order of K17 expression revealed no obvious relationships at the macro level between K17 expression and any immune cell type (Fig. 1c).

Based on the premise that not only the relative abundance of T cells, but also the distribution and spatial relationship between T-cell subpopulations and cancer cells reflect biological interactions, we next set out to develop a model to score immune cells in the spatial context of direct interaction, reflected by immune cells that overlapped or directly contact tumor cells (intratumoral immune cells) versus those present within 25 µm of the closest tumor cells (peritumoral immune cells), relative to the expression of K17 (Fig. 1d).

### K17 has profound effects on the PDAC immune microenvironment

Analytic algorithms were developed to count intratumoral and peritumoral immune cells (respectively those that directly contact tumor cells versus stromal immune cells located within 25 μm of the closest tumor cells, relative to K17 status). Immune cell counts were normalized relative to cell counts in K17-positive zones and results were ranked in order of increasing immune cell density ratios. In this analysis, immune cell ratios reflect differences in K17 negative versus K17-positive zones, rather than relative differences in overall immune cell counts across the entire tumor region.

**Fig. 2:**
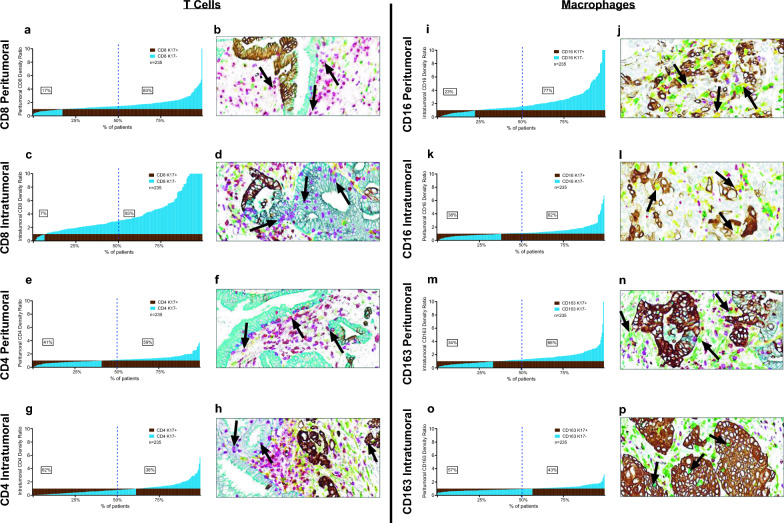
K17 impacts intratumoral and peritumoral T cells and macrophages. **a**–**h** T cell counts in peritumoral and intratumoral K17-positive and K17-negative regions. **a** Peritumoral CD8+ T cells. **c** Intratumoral CD8+ T cells. **e** Peritumoral CD4+ T cells. **g** Intratumoral CD4+ T cells. **i**–**p** Macrophage counts in peritumoral and intratumoral K17-negative regions relative to K17-positive regions. **i** Peritumoral CD16+ macrophages; **k** Intratumoral CD16+ macrophages; **m** peritumoral CD163+ macrophages; **o** intratumoral CD163+ macrophages. Representative mIHC images for each panel highlight intratumor and peritumor **b**, **d** CD8+ T cells (purple); **f**, **h** CD4+ T cells (red); **j**, **l** CD16+ macrophages (yellow) and; **n**, **p** CD163+ macrophages (green) relative to K17-positive tumor cells (brown) and K17-negative tumor cells (teal). Note that immune cell ratios are normalized to counts in K17-positive zones and relative height of the bars reflects the magnitude of differences between ratios in K17-negative versus K17-positive zones, not relative differences in overall immune cell counts

Cytotoxic T cells target tumor cells that expose tumor-specific antigens in various malignancies, including pancreatic ductal adenocarcinoma [7, 29, 44] and higher CD8+ T-cell density in tumor is generally associated with prolonged pancreatic cancer survival [10, 26, 55, 63]. Conversely, K17 has been associated with immune cell response in psoriasis as well as in basal cell skin cancer and in cervical carcinoma and is a negative prognostic biomarker in PDAC, suggesting that K17 might have some role in CD8+ T cell exclusion [60, 66]. Thus, to test for relationships between K17 expression the tumor inflammatory microenvironment, we analyzed intratumoral and peritumoral CD8+ T cells, CD4+ T cells, CD16+/CD163− tumor-targeting (M1) macrophages and CD16+/CD163+ tumor promoting (M2) immune cells ratios across all cases. CD8+ peritumoral T cells were more numerous in K17-negative areas than in K17+ areas *p* < 0.0001) in 83% of PDACs (Fig. 2a). Even more profoundly, intratumoral CD8+ T cell ratios were greater in K17-negative regions than in K17-positive regions in 93% of PDACs (*p* ≤ 0.0001) (Fig. 2c). Although the magnitude of the correlation with K17 was much less than seen for CD8+ T cells, peritumoral CD4+ T ratios were also greater in K17 negative areas for 59% of cases (Fig. 2e) but were increased in K17+ intratumoral areas in 62% of cases (Fig. 2g).

To uncover any relationships between K17 expression and macrophage distribution, we then analyzed the immune cell density of CD16+ macrophages and CD163+ macrophages across all cases. CD16+ cells were more abundant in K17 negative versus K17 positive peritumoral areas in 77% of cases (p < 0.0001) (Fig. 2i). Intratumoral CD16+ macrophages were more numerous in K17-negative tumor zones compared to the K17-negative regions in 62% of cases (p < 0.0001) (Fig. 2k). In peritumoral zones, CD163+ macrophages were more abundant in K17-negative zones in 66% of cases (p < 0.0001) (Fig. 2m). Conversely, intratumoral CD163+ macrophages were more numerous in K17-positive zones in 57% of cases (*p* ≤ 0.0001) (Fig. 2o). The relationships between CD16+ and CD163+ macrophages and K17 expression were independent of other clinicopathologic features, including tumor grade, pathological stage, treatment history, histologic variant, and mutational status (data not shown).

**Fig. 3:**
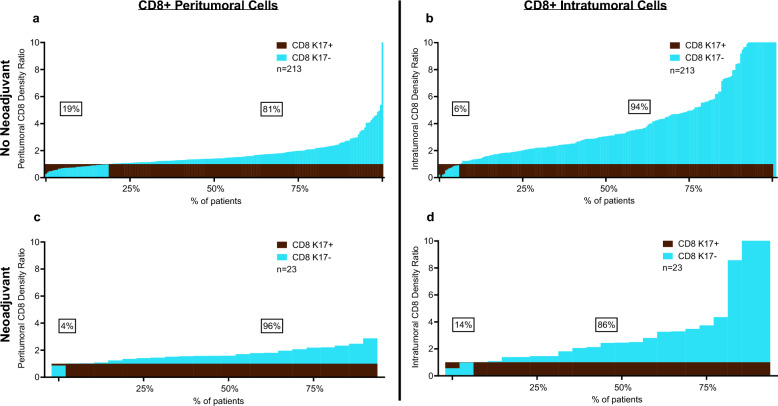
The impact of K17 on CD8+ T Cells is independent of neoadjuvant therapy. **a**, **b** Peritumoral and intratumoral CD8+ T cell density ratios in cases that did not receive neoadjuvant treatment and, **c**, **d** cases treated with neoadjuvant treatment

To explore changes in tumor-infiltrating immune cells in PDACs after neoadjuvant immunotherapy, we separate our cohort into two categories, including patients that received gemcitabine-based or 5-FU based neoadjuvant treatment (n = 23, 10%) versus those that did not receive any neoadjuvant treatment before surgery (n = 212, 90%). CD8+ T cell ratios were consistently greater in K17-negative peritumoral and intratumor zones, for both no-neoadjuvant and neoadjuvant treatment groups (Fig. 3a–d). These results suggest that neoadjuvant therapy has minimal impact on CD8+ T cell ratios in K17-negative versus K17-positive tumor zones.

**Fig. 4:**
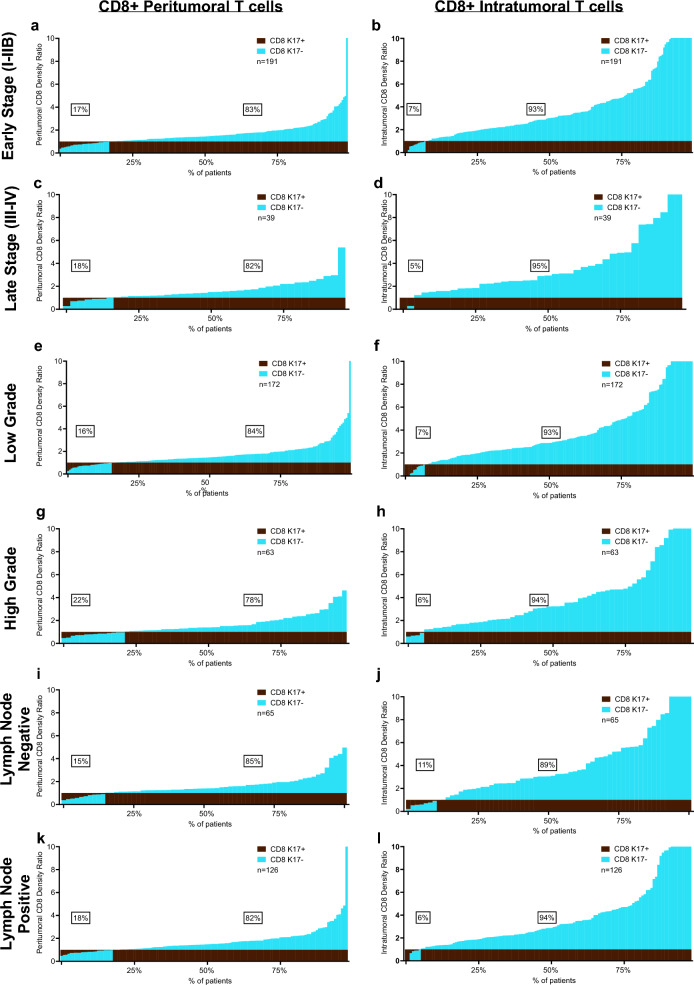
The impact of K17 on CD8+ T cells is independent of PDAC stage, grade, and lymph node status. Immune cell ratios in peritumoral and intratumoral K17-negative regions relative to K17-positive regions, ordered based on the density of immune cells in K17-positive zones. The inverse correlation between K17 expression and CD8+ peritumor and intratumoral T cells is independent of **a**–**d** stage, **e**–**h** tumor grade, and **i**–**l** Lymph node status

We next tested for relationships between tumor stage, grade and lymph node status and found that the inverse correlations between K17+ expression and CD8+ T cells are independent of each of these tumor-specific clinicopathologic variables (Fig. 4). Furthermore, CD8+ cell counts relative to K17 status were independent of tumor histologic subtype, including conventional, foamy cell, and large duct PDAC variants (Supp. Figure 6).

**Fig. 5:**
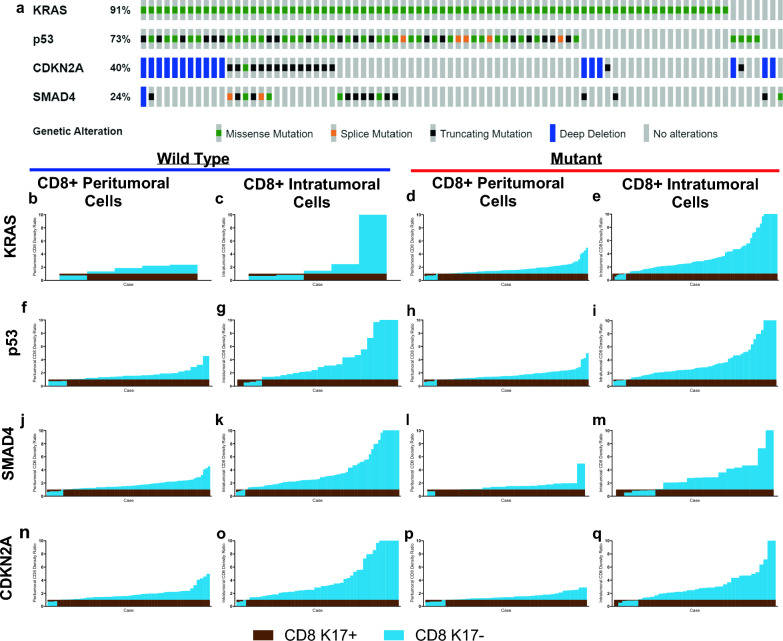
CD8+ T cells are increased in K17-negative regions, regardless of mutation status. Immune cell ratios in peritumoral and intratumoral K17-negative regions relative to K17-positive regions and mutational status of KRAS, p53, SMAD4, and CDKN2A. **a** OncoPrint [8, 12, 18] depicting the most frequently mutated genes in the KYT cohort. **b**–**q** Wild type versus mutant KRAS, p53, SMAD4, and CDKN2A

Several studies have reported that TP53 missense mutations lead to reduce the infiltration of cytotoxic CD8+ T cells and approximately 70% of all PDACs harbor TP53 gene mutations [28, 30, 39]. Furthermore, wild-type (WT) and mutant variants of p53 can modulate the antigen presentation machinery and can influence cytokine and chemokine secretion from the cancer cells, thereby impacting the immune TME [28]. We set out to elucidate the impact of the 4 most common mutations on the immune TME of PDAC based on the analysis PDACs from the KYT cohort that had undergone comprehensive genomic sequencing through the Precision Promise program of the Pancreatic Cancer Action Network [40, 41] (Fig. 5a). SIFT predicted that 96% of 137 KRAS mutations, 40% of 132 p53 mutations, 22% of 36 SMAD4 mutations, and 3% of 60 CDKN2A mutations were deleterious. Mutational status summary can be found in Supplementary Table 3. We divided our samples based on their genomic status into WT or Mutant for each gene and we found that regardless of the mutational landscape, the impact of K17 CD8+ T cell rations within the immune microenvironment was unchanged (Fig. 5b–q).

Thus, K17 expression correlates with major differences in the immune microenvironment, most notably through profound exclusion of CD8+ T cells that is independent of clinicopathologic features or tumor intrinsic variables, treatment history, tumor grade, pathological stage, lymph node status, histologic variant, and tumor mutational status.

## Discussion

Although K17 expression impacts gene expression, cell proliferation, and numerous other hallmarks of cancer, the impact of K17 on the immune response to PDAC has not previously been explored. In this study, we found that tumor cell expression of K17 expression impacts the PDAC microenvironment by shielding tumor cells from CD8+ T cells responses, while recruiting tumor promoting CD163+ (M2) macrophages, indicating that K17 impacts the immune response as a fundamental hallmark of aggression in PDAC. This work also provides a platform for image analysis of multiplexed immunohistochemical protocols that can efficiently analyze the immune composition of the cancer microenvironment.

PDAC is generally regarded as a “cold tumor” with a low T cell infiltration and low tumor mutation burden (TMB) with few neoantigens [56]. This has undermined attempts to develop immunotherapeutic approaches for PDAC. High levels of T cell infiltration, however, correlate with improved outcome in PDAC [19, 24], including CD8+ T Cells [36]. Interestingly, the proximity of CD8+ T cells to tumor cells in the PDAC TME correlates to longer patient survival [7]. Consistent with our previous works which showed that K17 expression in PDAC is associated with shorter survival our current findings also support the hypothesis that K17 blocks immune cell infiltration, with the most profound impact being on CD8+ T cells.

Therefore, advancing our understanding of the immunobiology of PDAC by identifying new targets and biomarkers predicting immunotherapy response is crucial for improving clinical outcomes. Although K17 impacts numerous hallmarks of cancer [5] and has been established as a defining biomarker of the basal molecular subtypes of pancreatic cancer [35, 46, 47] emerging clinical and research data support its mechanistic role in the regulation of the immune response [5, 6].

While the mechanisms underlying the immunomodulatory function of K17 are not fully understood and have not been explored in pancreatic cancer, previously published data suggests that K17 expression has been associated with the production of pro-inflammatory cytokines and chemokines that regulate the recruitment of immune cells to sites of inflammation and modulate immune responses. In basal cell carcinoma of the skin (BCC), increased K17 expression correlates with the expression of key pro-inflammatory chemokines such as CXCR3 andCXCL10, among others [5] and the genetic ablation of K17 delays BCC onset in mouse models that correlates with a global cytokine switch that differentially regulates T cell infiltration [14]. Furthermore, genetic knockout of K17 in a mouse papillomavirus (MmuPV1) model of cervical cancer results in rapid regression of papillomas and increased CD8+ T cell infiltration [59] whereas K17 expression promoted the expression of pro-inflammatory cytokines IFN ‐γ, CXCL9, CXC110, CXC111, TNF-α, and TGF-β, among others [65]. Similarly, in head and neck squamous cell carcinoma, the knockout of K17 in immunocompetent mouse model also resulted in tumor regression due to alterations in IFN ‐γ and CXCL9, and PD-L1 expression and increased CD8+ T-cell infiltration, thereby sensitizing tumors to immune-checkpoint blockade [59]. In PDAC, single-cell RNA seq analysis of 18 human biopsies identified a population of cells with high KRT17, CXCL8, and multiple other inflammatory cytokines, inducing the emergence of immunosuppressive immune cell populations [6]. Consistent with these observations, Raghavan et al. [44] identified TGF-β signaling by single-cell RNA seq analysis as a top-upregulated pathway in basal subtype PDAC, associated with exclusion of both CD8+ and CD4+ T cells from the tumor microenvironment. As K17 has been widely accepted as a marker of basal subtype (ref), this study further supports a possible link between K17 and TGF-β as a mechanism to drive the exclusion of CD8+ T cells.

Moreover, Bailey et al. [4] found that CD8A expression was low in PDACs, despite the high expression of HLA class I genes, presence of neoantigens and mutational load, and suggest this phenotype is associated with increase oxidative stress, mainly through the overexpression of iNOS/NOS2. Oxidative stress and hypoxia are known hallmarks of solid tumors, and iNOS can be induced by hypoxia [25]. Expression of K17 increased the Akt/mTOR/hypoxia-inducible factor (HIF)-1α pathway in osteosarcoma cells [62], and knockout of K17 restored this pathway expression.

Additionally, the role of K17 protein as an antigen in PDAC is unknown but may also play a role in modulating the PDAC TME, given K17 is normally expressed during embryogenesis but is silenced in mature somatic tissues [5]. Whether self-tolerance to K17 epitopes remains during PDAC tumor development is unknown, but anti-K17 Treg cells could play a role in dampening immune responses in the TME as well, as evidence suggest that during the formation of central tolerance, CD4+ T cells that react to self-derived epitopes are less likely to be deleted than CD8+ Tcells, and differentiate into Tregs [53].

Moreover, K17 has been shown to modulate pathways involved in immunity and inflammation, including the NF-κB and STAT3 signaling. Hobbs et al. [21] demonstrated that K17-dependent expression of an autoimmune regulator, Aire, is required for the timely onset of the Gli2-induced skin tumorigenesis in mouse model. The functional interaction between K17 and the heterogeneous nuclear ribonucleoprotein hnRNP K leads to activation of p65 (NF-kB) program in tumor-prone keratinocytes [21]. In the contact dermatitis mouse model, nuclear translocation of K17 facilitates the activation and nuclear translocation of signal transducer and activator of transcription 3 (STAT3) activating CCL20 production and CD8+ and CD4+ T cell trafficking to skin lesions [27]. Thus, the role of K17 in immune response in PDAC is complex and multifaceted. Further investigations are needed to fully understand the mechanisms underlying the immunomodulatory function of K17 in PDAC and to explore its potential as a biomarker of immune evasion a therapeutic target in cancer immunotherapy.

A multiparameter analysis of the immune landscape in PDAC revealed heterogeneous expression of immune checkpoint receptors in individual patients’ T cells and increased markers of CD8+ T cell dysfunction in the disease stage [51]. In vivo studies have also shown that blockade of IL-1β increased the numbers of tumor-infiltrating lymphocytes and CD8+ T-cell responses. Furthermore, Wang et al. [57] studied the role of K17 in cancer metastasis using an immunocompetent mice model and their results suggest that K17 confers resistance to immunotherapy. One mechanism through which K17 downregulates T cell infiltration could be through the suppression of CXCL9 production in macrophages through tumor cell-macrophage interactions. Other in vivo studies, also suggest that K17 expression suppressed T cell infiltration and enhanced neutrophil infiltration in in the tumor microenvironment of cervical cancers [58].

The delicate balance between the populations of CD4+ and CD8+ subsets determines whether the TME is anti- or pro-tumorigenic [48]. Notably, regulating the differentiation of naïve CD4+ T cells into Th1, Th2, Th17, Th9, Th22, and Tregs is essential for eliminating immunosuppressive restrictions from the tumor environment and boosting effector T-cell activity [22, 23, 32]. It is possible that the disruption of the correct ratio of these cell populations causes immune evasion in cancer and even the failure of several immune cell targeted therapies. We hypothesize that most CD4 T cells associated with K17-positive tumor areas are Tregs and that K17 contributes to PDAC growth by suppressing T cell infiltration. Although the mIHC panel described in this paper was not designed to identify CD4 T cells subsets, further studies to identify CD4 T cell subsets and their association with K17 expression in PDACs are ongoing in our lab.

K17 has a wide range of effects on the immune response in different tissues. For example, increased K17 expression upregulates the expression of multiple proinflammatory cytokines and chemokines, including IFN-γ, IL-22, and CXCL1, and plays an important role in the development of psoriasis. Whereas in models of head and neck cancer, the knockout of K17 gene expression slowed tumor growth and increased CD8+ T cell infiltrate in immunocompetent syngeneic C57/BL6 mice compared to parental MOC2 tumors [45, 57]. Here, we observed an inverse correlation between K17 and CD8+ T cells, as reported previously in other skin and allergic disease processes. Insight into the mechanism that underlie these effects may be inferred from previous studies that have linked K17 and CD8+ T cells in psoriasis and allergic contact dermatitis (ACD) [27, 60]. Providing further insight into the mechanisms through which K17 acts in ACD, it was found that K17 translocates into the nucleus of activated keratinocytes, facilitating activation of STAT3 and downstream CCL20 production as well as T cell trafficking. Our lab previously reported that the soluble form of K17 undergoes nuclear translocation and serves as a nuclear shuttle of p27 [15]. Thus, it is possible that similar mechanisms may have a role in the immune response to PDAC. M2 macrophages contribute to chronic inflammation, cancer cell stemness, desmoplasia, immune suppression, and metastasis in PDAC, highlighting their importance in pancreatic cancer [42]. Our observations that CD163+ (M2) macrophages are more numerous in K17-positive intratumoral areas are consistent with previous studies in colorectal cancer [61] and align with work depicting CD163 CD+ T cells as promoter of biologic aggression in pancreatic cancer [64].

In conclusion, our data support the hypotheses that K17 shields tumor cells from CD8+ T cells and recruits tumor promoting CD163+ M2 macrophages, indicating that K17 fundamentally impacts the immune response to PDAC. These effects are independent of neoadjuvant treatment, clinical pathologic features, or PDAC mutational status, suggesting that the interactions between K17 and immune cell responses in cancer are robust and could be important in both early stage and advanced stage disease. Beyond our exploration of tumor and immune cell interactions that are impacted by K17, the development of a platform for image analysis of multiplexed immunohistochemical protocols may also be applicable for the analysis of immune composition for solid tumors of other anatomic sites. Further studies are still needed to uncover how K17 expression facilitates evasion from immune surveillance, and to identify new druggable targets, relative to K17 status, that could enhance the efficacy of immunotherapy for PDAC. Whether K17 could also be used as a biomarker to identify subgroups of PDAC patients who may benefit from immunotherapy or could be therapeutically targeted to restore the efficacy of the innate immune response against PDAC should also be subjects of future research.

## Conclusions

K17 expression shields tumor cells from CD8+ T cells and recruits tumor promoting CD163+ M2 macrophages, indicating that K17 fundamentally impacts the immune response to PDAC. These effects are independent of neoadjuvant treatment, clinical pathologic features, or PDAC mutational status, suggesting that the interactions between K17 and immune cell responses in cancer are robust and could be important in both early stage and advanced stage disease. Therefore, targeting K17-mediated immune effects on the immune system could potentially restore the innate immunologic response to PDAC and might provide novel immunotherapeutic approaches for this devastating disease.

### Supplementary Information

Supplementary Material 1: Supplementary Fig. 1. Example of region of interest with manual dot annotation on the training set is illustrated. Expert pathologists manually placed dots on each cell over the ROIs a. Original mIHC ROI from PDAC testing case retrieved from QuIP; and ground truth (Superpixel Label and dot annotation) overlaid on original Image of b. K17 positive tumor cells (brown); c. K17 negative tumor cells (panCK in teal); d. CD163+ macrophages in green; e. CD16+ macrophages; f. CD8+ T cells in purple g. CD4+ T cells in red. Supplementary Fig. 2. Example of Region of interest with ground truth annotation. (A) Original mIHC ROI from PDAC testing case retrieved from QuIP (B) ground truth (Superpixel Label and dot annotation) overlaid on original Image. The following colors represent different stains: (black = CD4, purple = CD8, yellow = CD16, green = CD163, brown = K17+, Teal = K17. Supplementary Fig. 3. Immune cell density at different distances from the closest tumor nest margin. a. Bar graph depicting mean CD4+ cell density and standard deviation for 8 cases; b. CD8+ cell mean density; c. CD16+ and; d. CD163+ cells. Supplementary Fig. 4. High K17 expression is correlated with shorter survival in patients. Kaplan–Meier curves for the overall survival analysis of K17 from PDAC cases of all stages. P values were calculated using the log-rank test. HR, hazard ratio; K17, keratin 17; PDAC, pancreatic ductal adenocarcinoma. Supplementary Fig. 5. Flowchart of Multiplex Immunohistochemical Whole Slide Image Analysis Pipeline mIHC: multiplex immunohistochemistry, WSI: whole slide image, ROI: Region of Interest. Supplementary Fig. 6. There are more CD8+ T cells in K17-negative regions, regardless of histologic variant. Immune cell ratios in peritumoral and intratumoral K17-negative regions relative to K17-positive regions, ordered based on the density of immune cells in K17-positive zones. a–c. Peritumoral CD8+ T cell density ratios in conventional, foamy cell, and large duct PDAC variant cases. d–f. Intratumoral CD8+ T cell density ratios in conventional, foamy cell, and large duct PDAC variant cases. g–I. Representative H&E photomicrographs of conventional, foamy cell, and large duct PDAC variants, respectively. Supplementary Table 1. List of targeted populations, antibodies, incubation times, chromogens, and localization of each marker used in mIHC staining. Supplementary Table 2. Model Validation. Supplementary Table 3. Mutational status.

## Data Availability

The datasets generated and/or analyzed during the current study are not publicly available due to IRB regulations but are available from the corresponding author on reasonable request. All required pipelines for digital image processing and related computational manuals are available at https://github.com/SBU-BMI/shroyer_lab_workflow to expand biomarker-based discovery and deployment in oncoimmunology research and improved ability to stratify and monitor patients receiving diverse immune based therapeutics.

## Abbreviations

ACD: Allergic contact dermatitis
AJCC: American Joint Committee on Cancer
CITI: Collaborative Institutional Training Initiative
CNN: Convolutional neural network
FFPE: Formalin-fixed paraffin-embedded
IRB: Institutional Review Board
K17: Keratin 17
mIHC: Multiplexed immunohistochemistry
PanCAN: Pancreatic Cancer Action Network
panCK: Pancytokeratin
PDAC: Pancreatic ductal adenocarcinoma
ROI: Region of interest
scRNA: Single-cell RNA
SLIC: Simple linear iterative clustering
TMB: Tumor mutation burden
TME: Tumor microenvironment
WHO: World Health Organization
WSI: Whole slide image
WT: Wild type
XSEDE: EXtreme Science and Engineering Discovery Environment
