# Supplementary material for: Keratin 17 modulates the immune topography of pancreatic cancer
Source: J Transl Med. 2024 May 10;22:443. doi: 10.1186/s12967-024-05252-1 (PMC11087249; doi:10.1186/s12967-024-05252-1)
Supplement: Supplementary file 1 — Supplementary Material 1: Supplementary Fig. 1. Example of region of interest with manual dot annotation on the training set is illustrated. Expert pathologists manually placed dots on each cell over the ROIs a. Original mIHC ROI from PDAC testing case retrieved from QuIP; and ground truth (Superpixel Label and dot annotation) overlaid on original Image of b. K17 positive tumor cells (brown); c. K17 negative tumor cells (panCK in teal); d. CD163+ macrophages in green; e. CD16+ macrophages; f. CD8+ T cells in purple g. CD4+ T cells in red. Supplementary Fig. 2. Example of Region of interest with ground truth annotation. (A) Original mIHC ROI from PDAC testing case retrieved from QuIP (B) ground truth (Superpixel Label and dot annotation) overlaid on original Image. The following colors represent different stains: (black = CD4, purple = CD8, yellow = CD16, green = CD163, brown = K17+, Teal = K17. Supplementary Fig. 3. Immune cell density at different distances from the closest tumor nest margin. a. Bar graph depicting mean CD4+ cell density and standard deviation for 8 cases; b. CD8+ cell mean density; c. CD16+ and; d. CD163+ cells. Supplementary Fig. 4. High K17 expression is correlated with shorter survival in patients. Kaplan–Meier curves for the overall survival analysis of K17 from PDAC cases of all stages. P values were calculated using the log-rank test. HR, hazard ratio; K17, keratin 17; PDAC, pancreatic ductal adenocarcinoma. Supplementary Fig. 5. Flowchart of Multiplex Immunohistochemical Whole Slide Image Analysis Pipeline mIHC: multiplex immunohistochemistry, WSI: whole slide image, ROI: Region of Interest. Supplementary Fig. 6. There are more CD8+ T cells in K17-negative regions, regardless of histologic variant. Immune cell ratios in peritumoral and intratumoral K17-negative regions relative to K17-positive regions, ordered based on the density of immune cells in K17-positive zones. a–c. Peritumoral CD8+ T cell density ratios in conventional, foamy cell, [file 12967_2024_5252_MOESM1_ESM.docx]

**
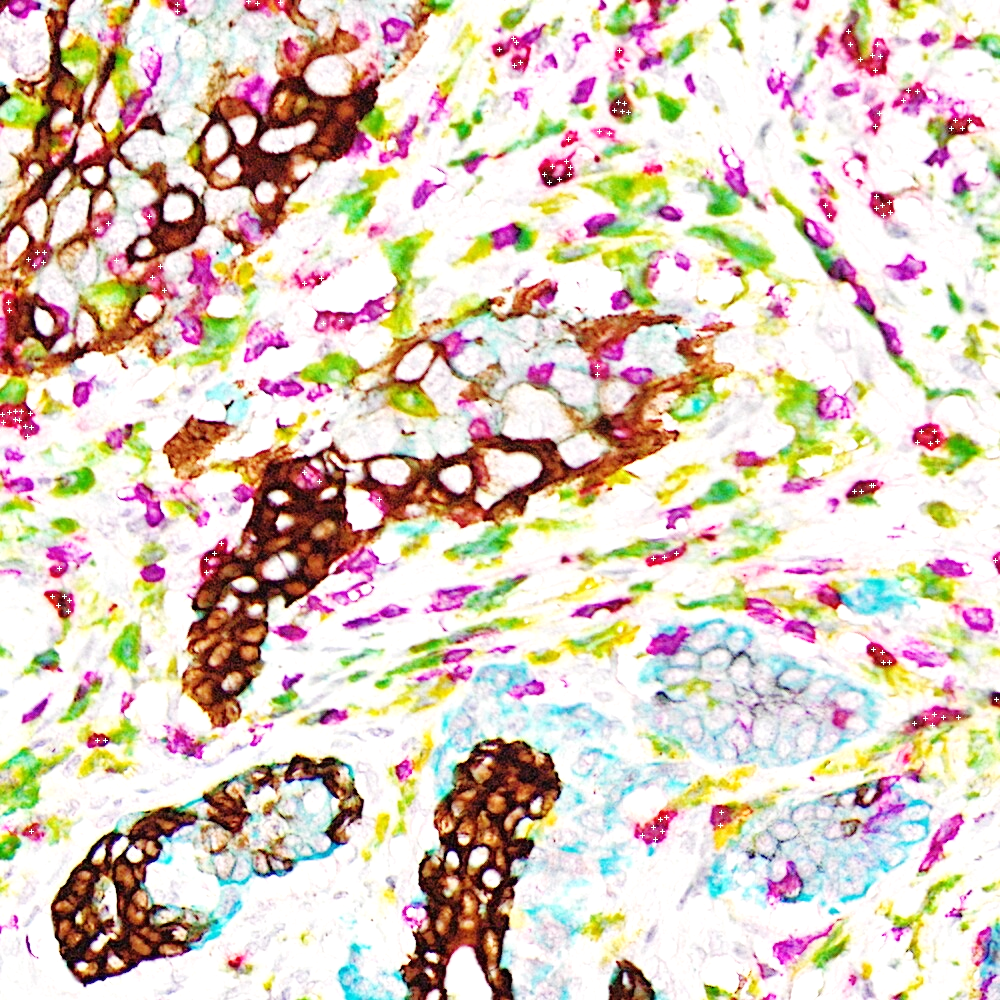

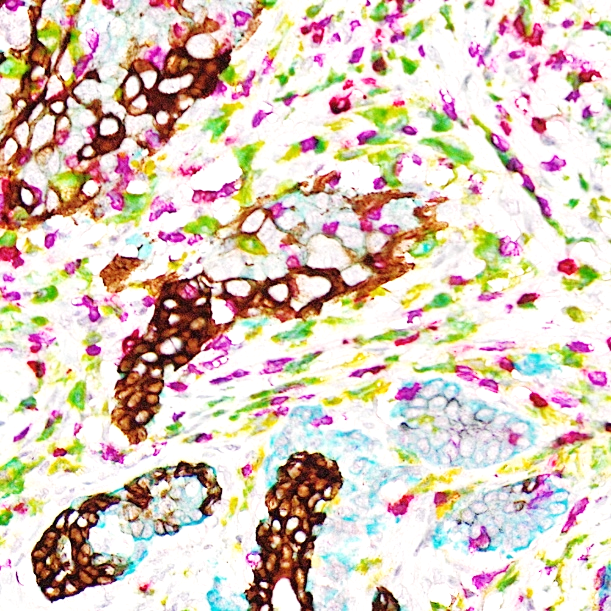

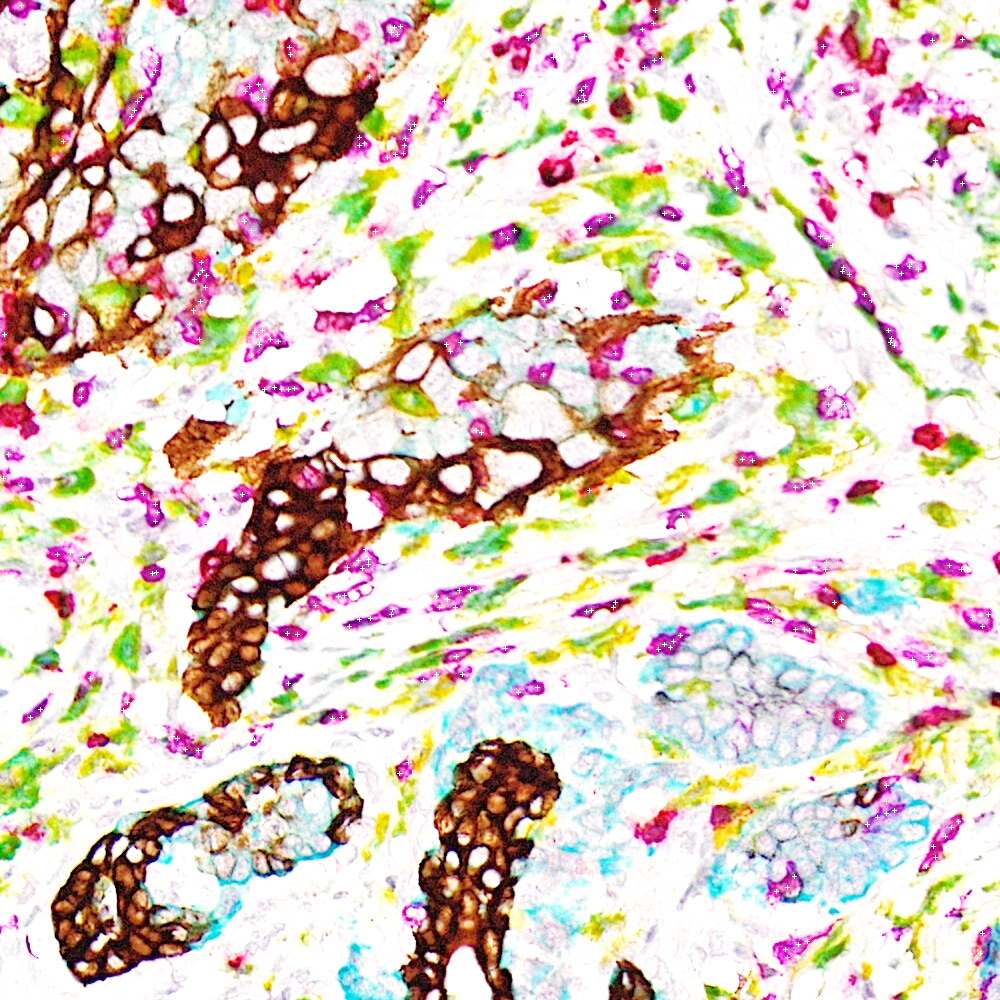

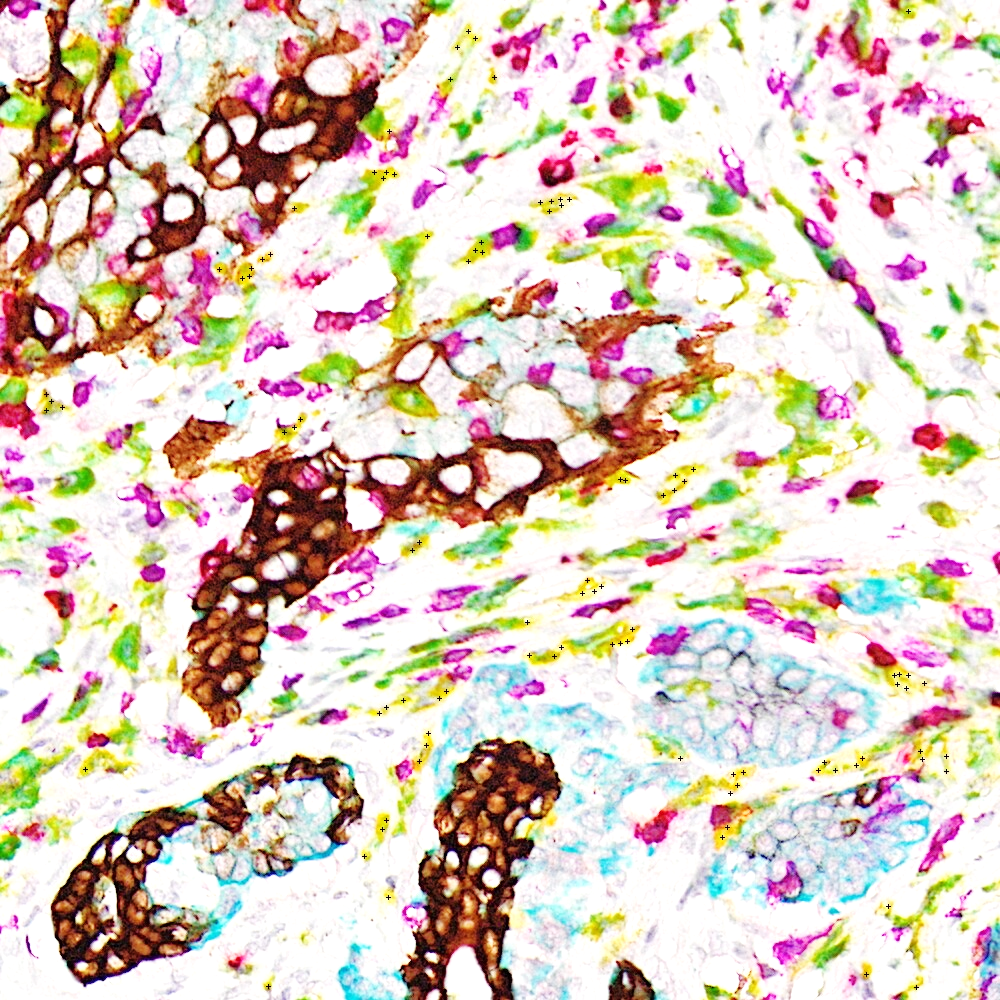

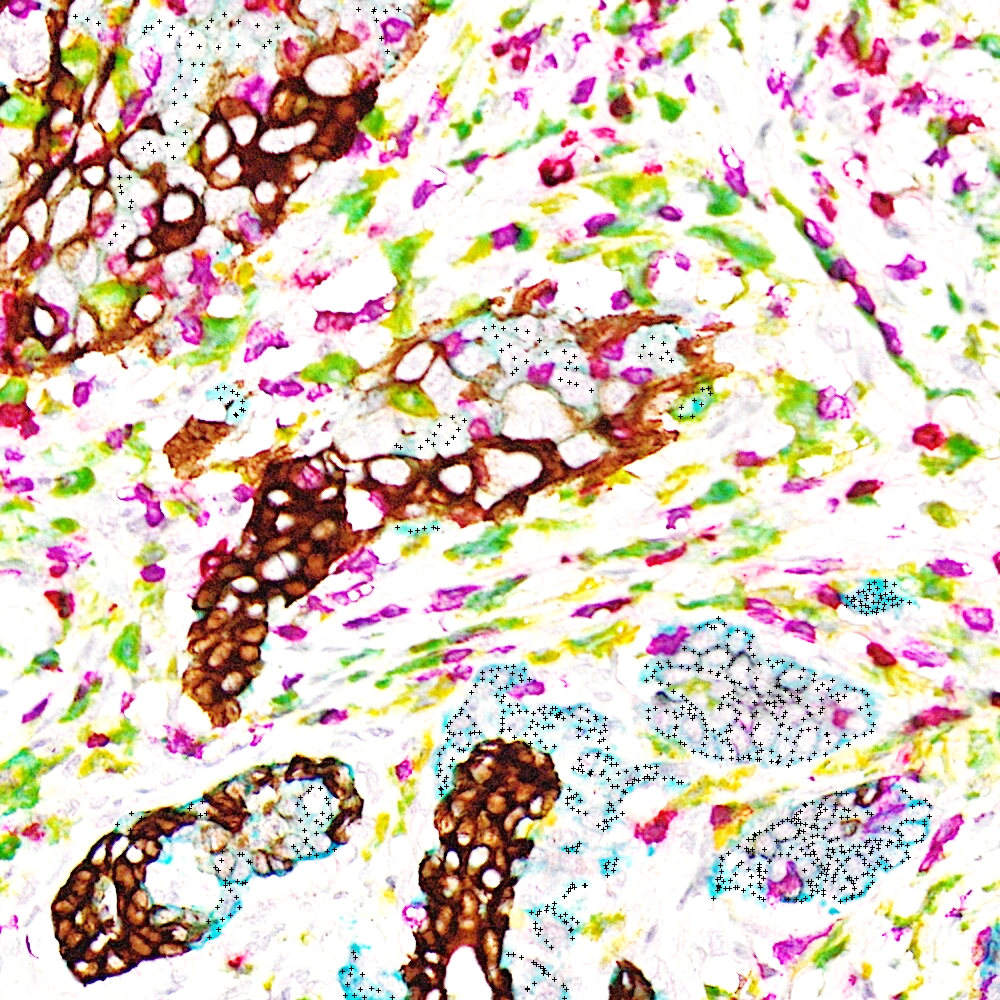

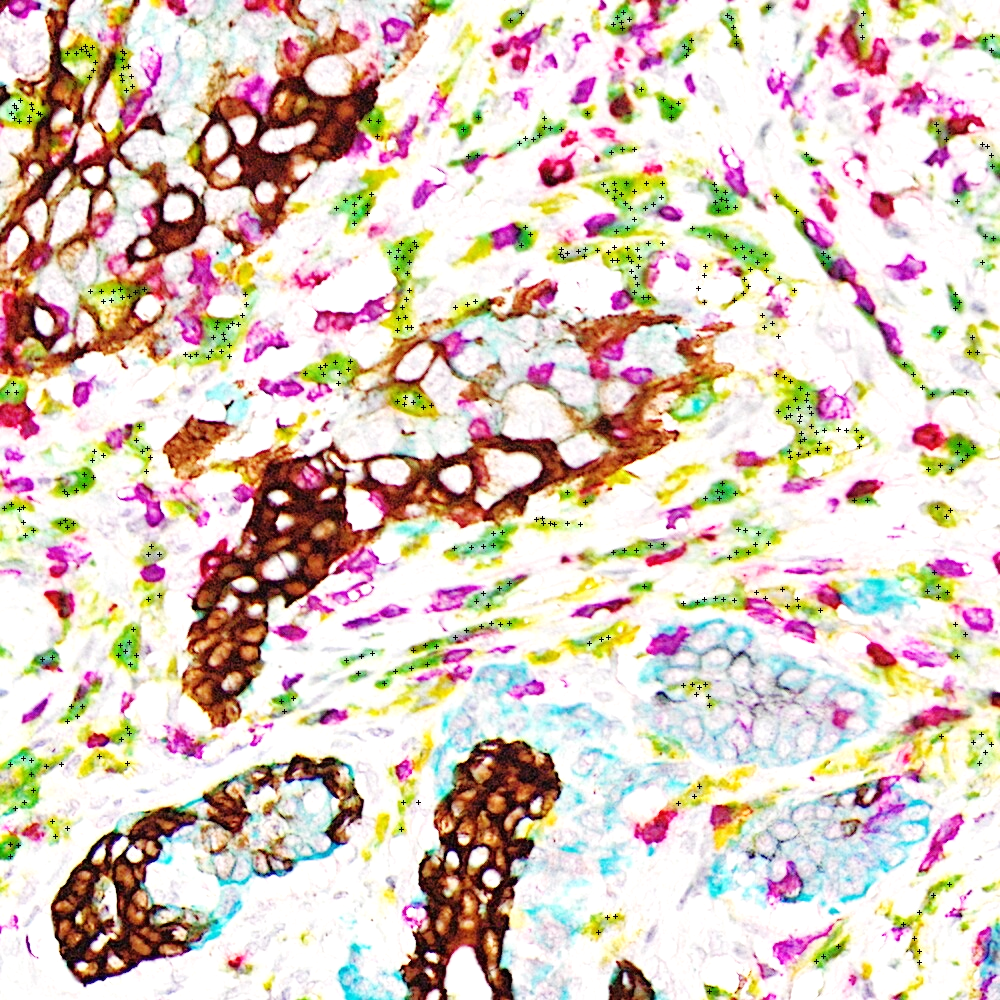

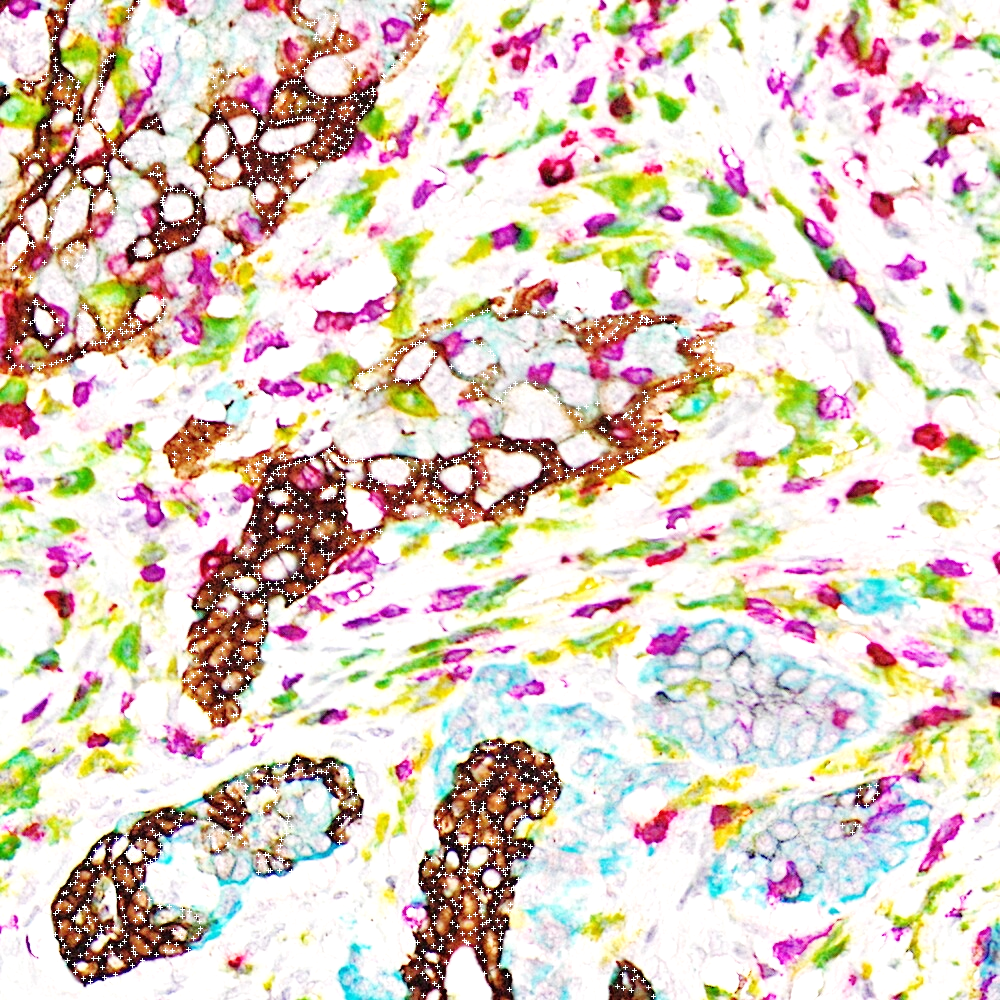
**

**c**

**d**

**g**

**c**

**b**

**e**

**g**

**e**

**f**

**a**

**f**

**b**

**d**

**b**

**Supplementary Figure 1.** **Example of Region of interest with manual dot annotation on the training set is illustrated.** Expert pathologists manually placed dots on each cell over the ROIs **a.** Original mIHC ROI from PDAC testing case retrieved from QuIP; and ground truth (Superpixel Label and dot annotation) overlaid on original Image of **b.** K17 positive tumor cells (brown); **c.** K17 negative tumor cells (panCK in teal); **d.** CD163+ macrophages in green; **e.** CD16+ macrophages; **f.** CD8+ T cells in purple **g.** CD4+ T cells in red.

**a** **b**

**
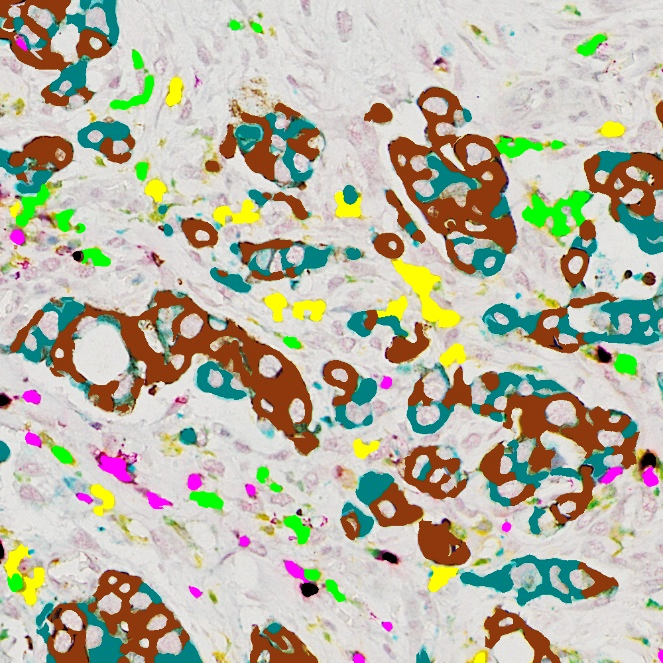

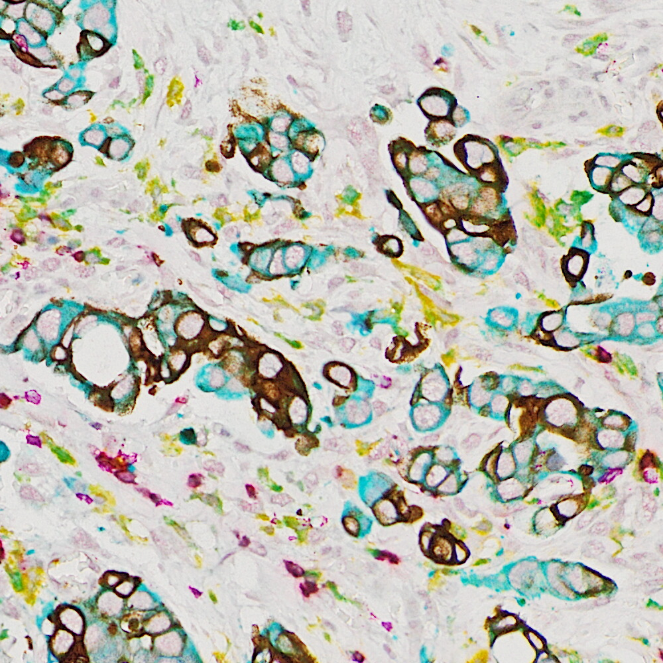
****Supplementary Figure 2.** **Example of Region of interest with ground truth annotation.** **a.** Original mIHC ROI from PDAC testing case retrieved from QuIP; **b.** ground truth (Superpixel Label and dot annotation) overlaid on original Image. The following colors represent different stains: (black = CD4, purple = CD8, yellow = CD16, green = CD163, brown = K17+, Teal = K17-).

**Supplementary Figure 3. Immune cell density at different distances from the closest tumor nest margin. a.** Bar graph depicting mean CD4 + cell density and standard deviation for 8 cases**; b.** CD8+ cell mean density; **c.** CD16+ and **d.** CD163+ cells.

**
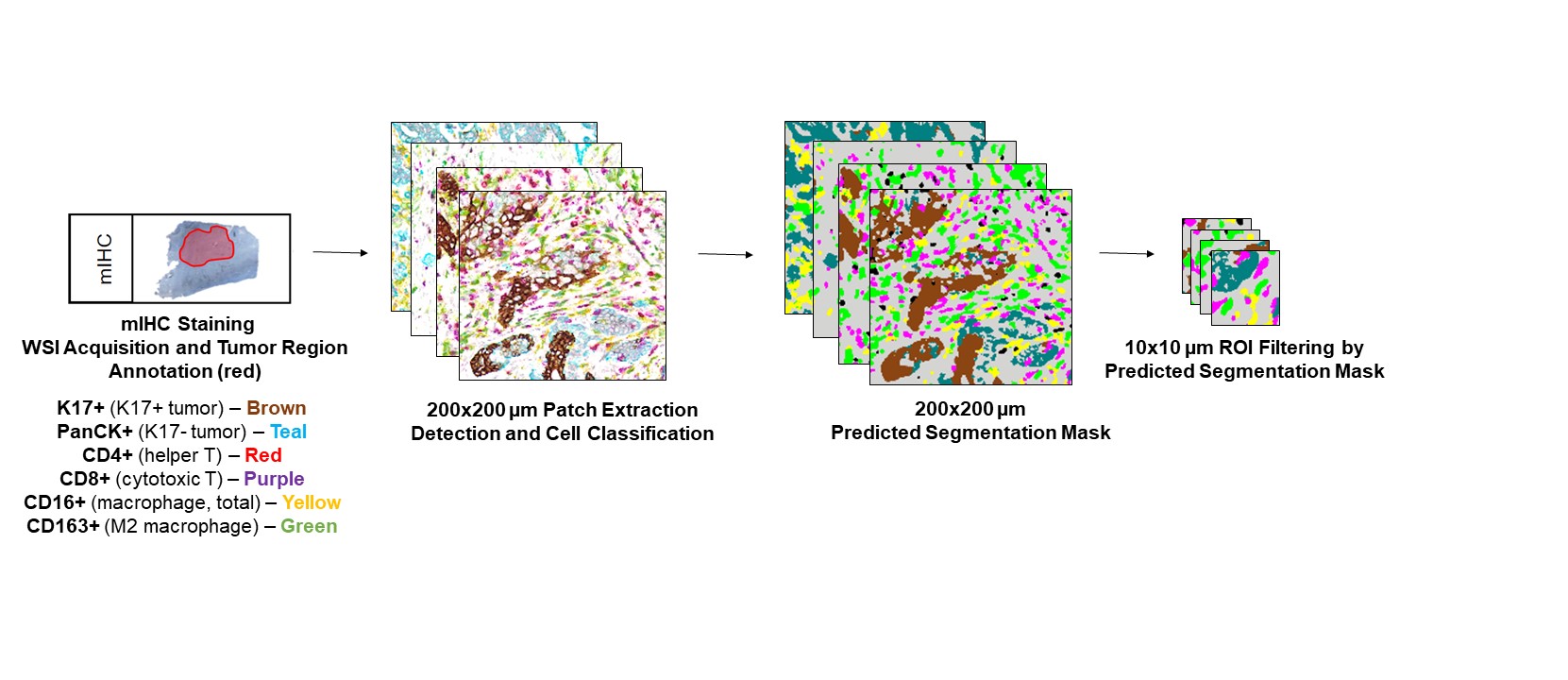
**

**Supplementary Figure 4. Flowchart of Multiplex Immunohistochemical Whole Slide Image Analysis Pipeline** mIHC: multiplex immunohistochemistry, WSI: whole slide image, ROI: Region of Interest.

**Supplementary Figure 5.** High K17 expression is correlated with shorter survival in patients. Kaplan-Meier curves for the overall survival analysis of K17 from PDAC cases of all stages. P values were calculated using the log-rank test. HR, hazard ratio; K17, keratin 17; PDAC, pancreatic ductal adenocarcinoma.


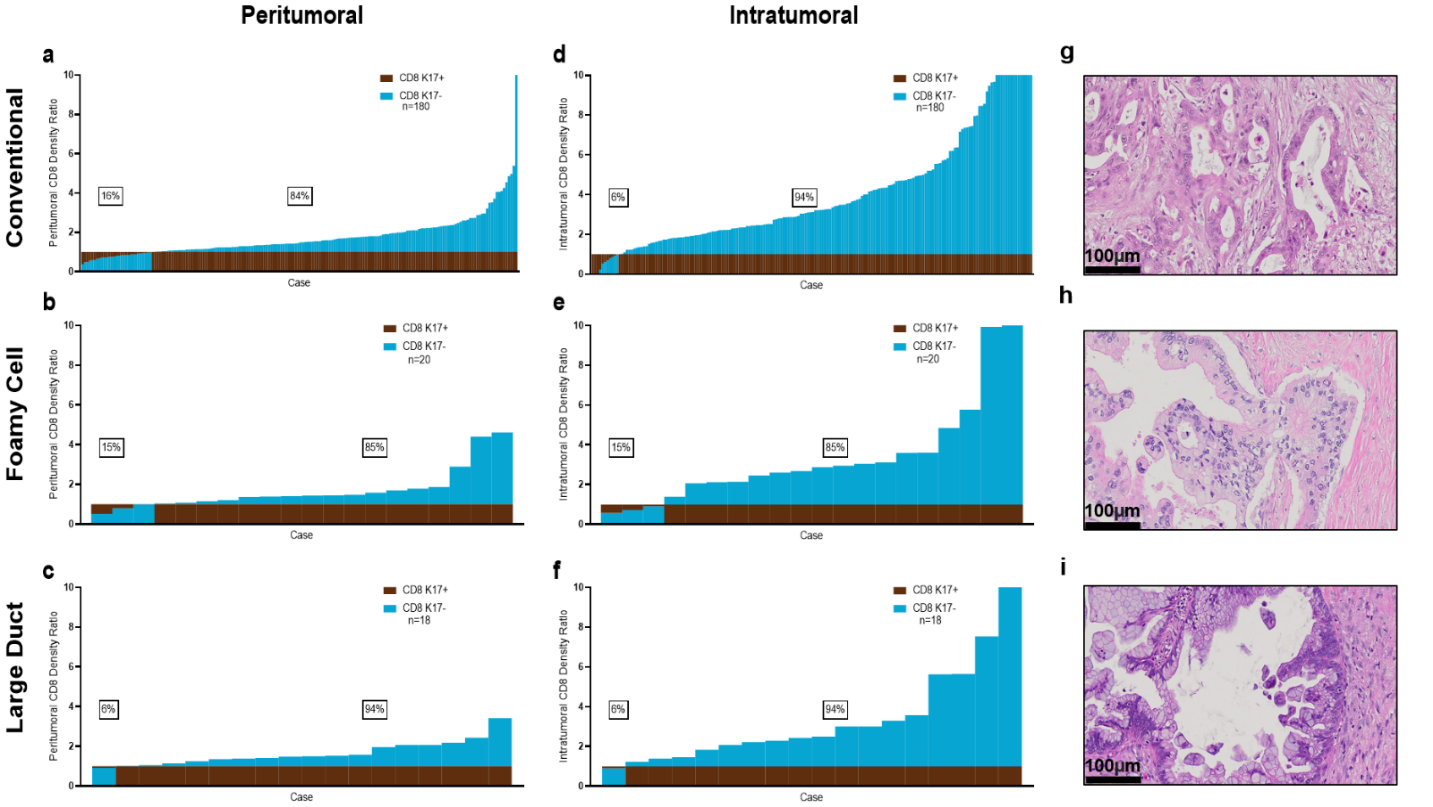


**Supplementary Figure 6. There are more CD8+ T cells in K17-negative regions, regardless of histologic variant.** Immune cell ratios in peritumoral and intratumoral K17-negative regions relative to K17-positive regions, ordered based on the density of immune cells in K17-positive zones. **a-c**. Peritumoral CD8+ T cell density ratios in conventional, foamy cell, and large duct PDAC variant cases. **d-f**. Intratumoral CD8+ T cell density ratios in conventional, foamy cell, and large duct PDAC variant cases. **g-I.** Representative H&E photomicrographs of conventional, foamy cell, and large duct PDAC variants, respectively.

**Supplementary** **Table 1.** List of targeted populations, antibodies, incubation times, chromogens, and localization of each marker used in mIHC staining.

| **Staining Order** | **Target** | **Primary Antibody** | **Clone** | **Vendor** | **Incubation** | **Dilution** | **Detection** | **Chromogen** |
| --- | --- | --- | --- | --- | --- | --- | --- | --- |
| 3 | K17+ PDAC Cells | *anti-****Cytokeratin 17*** *Rabbit Monoclonal Primary Antibody* | *SP95* | Roche | 40 min | Prediluted | DISCOVERY ChromoMap DAB (RUO) | Brown |
| 2 | CD8+ T cells T Cells | *CONFIRM anti-****CD8*** *Rabbit Monoclonal Primary Antibody* | *SP57* | Roche | 20 min | Prediluted | DISCOVERY Purple Kit (RUO) | Purple |
| 1 | M2 Macrophages | ***CD163*** | *MRQ-26* | Roche | 40 min | Prediluted | DISCOVERY Green HRP Kit (RUO) | Green |
| 4 | All Macrophages | ***CD16*** *Rabbit Monoclonal Primary Antibody* | *SP175* | Roche | 24 min | Prediluted | DISCOVERY Yellow Kit (RUO) | Yellow |
| 6 | All Ductal Epithelial Cells | *anti-****Pan Keratin*** *Primary Antibody* | *AE1/AE3/PCK26* | Roche | 24 min | Prediluted | DISCOVERY Teal HRP Kit (RUO) | Teal |
| 5 | Helper T Cells | *CONFIRM anti-****CD4*** *Rabbit Monoclonal Primary Antibody* | *SP35* | Roche | 32 min | Prediluted | DISCOVERY Silver Kit (RUO) | Black |

**Supplementary** **Table 2. Model Validation**

|  | **Metric** | **CD4** | **CD8** | **CD16** | **CD163** | **K17+** | **PanCk** |
| --- | --- | --- | --- | --- | --- | --- | --- |
| 5um | PPV | 0.9 | 0.94 | 0.96 | 0.76 | 0.98 | 0.96 |
|  | Recall | 0.67 | 0.88 | 0.66 | 0.96 | 0.99 | 0.97 |
|  | F1-score | 0.77 | 0.91 | 0.78 | 0.85 | 0.99 | 0.97 |
| 10um | PPV | 0.96 | 0.95 | 0.96 | 0.77 | 0.98 | 0.97 |
|  | Recall | 0.78 | 0.89 | 0.68 | 0.97 | 0.99 | 0.99 |
|  | F1-score | 0.87 | 0.92 | 0.79 | 0.86 | 0.99 | 0.97 |

**Supplementary** **Table 3. Mutational status**

| **Gene** | **Type** | **SIFT Prediction** | **Number of patients (%)** |
| --- | --- | --- | --- |
| KRAS | Missense | Deleterious | 132 (96.4%) |
| KRAS | Missense | Unknown | 1 (0.6%) |
| KRAS | CNA | N/A | 4 (3%) |
| CDKN2A | Missense | Deleterious | 2 (3.3%) |
| CDKN2A | TRUNC | N/A | 28 (47%) |
| CDKN2A | CNA | N/A | 29 (48.3%) |
| CDKN2A | Missense | Tolerated | 1 (1.4%) |
| p53 | Missense | Deleterious | 53 (52%) |
| p53 | Missense | Unknown | 7 (6.9%) |
| p53 | SPLICE | N/A | 13 (12.7%) |
| p53 | TRUNC | N/A | 29 (28.4%) |
| SMAD4 | Missense | Deleterious | 4 (12.5%) |
| SMAD4 | Missense | Unknown | 2 (6.3%) |
| SMAD4 | CNA | N/A | 3 (9.4%) |
| SMAD4 | FUSION | N/A | 1 (3%) |
| SMAD4 | SPLICE | N/A | 3 (9.4%) |
| SMAD4 | TRUNC | N/A | 19 (59.4%) |
